# Benign Regulation of the Astrocytic Phospholipase A_2_-Arachidonic Acid Pathway: The Underlying Mechanism of the Beneficial Effects of Manual Acupuncture on CBF

**DOI:** 10.3389/fnins.2019.01354

**Published:** 2020-02-04

**Authors:** Ning Ding, Jing Jiang, Huiling Tian, Shun Wang, Zhigang Li

**Affiliations:** ^1^Department of Acupuncture, Guang’anmen Hospital, China Academy of Chinese Medical Sciences, Beijing, China; ^2^School of Nursing, Beijing University of Chinese Medicine, Beijing, China; ^3^School of Acupuncture, Moxibustion and Tuina, Beijing University of Chinese Medicine, Beijing, China

**Keywords:** manual acupuncture, Alzheimer’s disease, astrocyte, hippocampus, phospholipase A_2_, arachidonic acid, cerebral blood flow

## Abstract

**Background:**

The astrocytic phospholipase A_2_ (PLA_2_)-arachidonic acid (AA) pathway is crucial in understanding the reduction of cerebral blood flow (CBF) prior to cognitive deterioration. In complementary and alternative medicine, manual acupuncture (MA) is used as one of the most important therapies for Alzheimer’s disease (AD). The beneficial effects of MA on CBF were reported in our previous study. However, the underlying molecular mechanism remains largely elusive.

**Objective:**

To investigate the effect of MA on the astrocytic PLA_2_-AA pathway in SAMP8 mice hippocampi.

**Methods:**

SAMP8 mice were divided into the SAMP8 control (Pc) group, the SAMP8 MA (Pm) group and the SAMP8 donepezil (Pd) group. SAMR1 mice were used as the SAMRl control (Rc) group. Mice in the Pd group were treated with donepezil hydrochloride at 0.65 μg/g. In the Pm group, MA was applied at Baihui (GV20) and Yintang (GV29) for 20 min. The above treatments were administered once a day for 26 consecutive days. The Morris water maze was applied to assess spatial learning and memory. Immunofluorescence staining, western blot and liquid chromatography-tandem mass spectrometry were used to investigate the expression of related proteins and measure the contents of the metabolic intermediates of the PLA_2_-AA pathway.

**Results:**

Compared with that in the Rc group, the escape latency in the Pc group significantly increased (*p* < 0.01); whereas, the platform crossover number and percentage of time and swimming distance in the platform quadrant decreased (*p* < 0.01). The hippocampal expression of PLA_2_, cyclooxygenase-1, cytochrome P450 proteins 2C23 and the levels of AA, prostaglandin E_2_ and epoxyeicosatrienoic acids of the Pc group was drastically higher than that in the Rc group (*p* < 0.01). These changes were reversed by MA and donepezil (*p* < 0.01 or *p* < 0.05).

**Conclusion:**

MA can effectively improve the learning and memory abilities of SAMP8 mice and has a negative regulatory effect on the PLA_2_-AA pathway. We propose that the increase of the arterial tone, which is induced by the inhibition of vasodilatory pathway, may be a reason for the beneficial effect of MA on CBF.

## Introduction

Alzheimer’s disease (AD) is a neurodegenerative disease of the central nervous system, characterized by a progressive loss of memory and a cognitive impairment. The main clinical manifestations include memory disorders, aphasia, apraxia, agnosia, loss of discernment capacity, and personality and behavioral changes. As the most common cause of dementia, AD accounts for 60–70% of patients with dementia (Di Marco et al., 2015; Chen et al., 2016) and is a highly prevalent disease, that has a high morbidity rate and high medical costs. Research shows that the global estimates of costs for dementia will be US $2.54 trillion in 2030 and US $9.12 trillion in 2050 (Jia et al., 2018). With the aging of population, the increasingly heavy financial and social burden imposed by AD, renders it a public health problem that require urgent solutions (Jones et al., 2017; Lane et al., 2018).

The pathogenesis of AD is thought to be a complex process associated with amyloid-β (Aβ) deposition, tau phosphorylation, oxidative stress and neuroinflammation. Among the pathological changes, cerebral vascular dysfunction plays an important role in AD early onset and development (Marchesi, 2011; Lourenco et al., 2017; Bennett et al., 2018). A series of imaging studies indicated that the cerebral blood flow (CBF) is significantly changed in AD patients (Okonkwo et al., 2014; Liu et al., 2015; van de Haar et al., 2016). As the first step in AD development, a decrease in CBF occurs before the appearance of clinical symptoms, which suggests its potential inducing role of AD pathological changes, including the expression of amyloid precursor protein (APP) and tau phosphorylation (Koike et al., 2010; Wang et al., 2010; Salminen et al., 2017). These sustained CBF changes have been associated with cognitive impairment (Hanyu et al., 2010; Leeuwis et al., 2017; Nielsen et al., 2017), and are disease specific (Abdi et al., 2012; Gao et al., 2013; Le Heron et al., 2014) that precede the other AD clinical symptoms (Hays et al., 2016). CBF reduction is a sensitive marker of AD early perfusion disorders (Lacalle-Aurioles et al., 2014). In particular, ischemia and anoxia, that are caused by a CBF decrease, can directly induce neuronal injury, synaptic dysfunction and promote Aβ accumulation by elevating the expression of the APP and reducing Aβ clearance. These events result in neural injuries and neurological disorders, that start the neurodegenerative process (Zlokovic, 2011; Sagare et al., 2012; Nelson et al., 2016). This compelling evidence suggests that the abnormality of the cerebrovascular function is one of the basic pathological changes and the key point of AD pathogenesis (Jellinger, 2008; Kelleher and Soiza, 2013).

Additionally, neurovascular injuries and dysfunctions of their signaling networks play a pivotal role in CBF reduction (Kuchibhotla et al., 2009; Iadecola, 2013; Delekate et al., 2014). For instance, astrocytes are of crucial importance for the cerebral hemodynamic stability and regulation and are also an essential factor in neurovascular coupling (McCaslin et al., 2011; Tarantini et al., 2017). The neurotransmitter pathway, mediated by glutamate, can promote calcium release from the endoplasmic reticulum, resulting in astrocytes increased intracellular calcium (Attwell et al., 2010). Calcium fluxes initiate the phospholipase A_2_ (PLA_2_)-arachidonic acid (AA) pathway (Zonta et al., 2003). PLA_2_ plays a key part in CBF molecular mechanism by regulating the vascular pathway (He et al., 2012). When the intracellular PLA_2_ is activated, membrane phospholipids are hydrolyzed into AA (Yagami et al., 2014; Leslie, 2015), which can further transform into related metabolic intermediates under the action of related enzymes. Through the COX, CYP2C, and CYP2J pathways, AA can be further transformed into PGE_2_ and EETs (Spector, 2009; Powell and Rokach, 2015). The vasoactive substances PGE_2_ and EETs can activate the potassium channels in the vascular smooth muscle cells after release from astrocytes end foot, resulting in vasodilatation and CBF increase (Attwell et al., 2010; Howarth, 2014; Zhang H. et al., 2017). These neurovascular pathways are crucial in understanding the neuropathic cascades that precede cognitive deterioration and dementia development (de la Torre, 2010), and have therefore, the potential to become new therapeutic targets in AD treatment (Zlokovic, 2005; van Norden et al., 2012; Acosta et al., 2017).

As one of the most important therapies in complementary and alternative medicine, acupuncture plays an active role in the clinical efforts against AD. Acupuncture can noticeably improve cognitive function and the quality of life of AD patients, by providing reduced side effects and safety (Zhou et al., 2015, 2017; Jia et al., 2017). Acupuncture is effective in improving the sleep quality of elderly patients with dementia (Kwok et al., 2013; Simoncini et al., 2015). Studies have partially clarified the mechanisms by which acupuncture can prevent AD through anti-inflammatory (Ding et al., 2017), anti-oxidative (Sutalangka et al., 2013), and anti-apoptotic effects (Guo et al., 2015, 2016). Moreover, acupuncture can modulate the functional activity and connectivity of specific cognition-related areas of the brain in AD patients (Wang et al., 2012, 2014; Liang et al., 2014; Zheng et al., 2018). However, the effect of manual acupuncture (MA) on CBF in AD therapy and the specifics of its molecular mechanisms are still not well known. Our previous study showed, for the first time, that MA can effectively enhance CBF in the prefrontal lobes and hippocampi of senescence-accelerated prone 8 (SAMP8) mice by MRI. We proposed that CBF increase could be an important mechanism of MA beneficial cognitive effects in AD (Ding et al., 2019). Considering that CBF plays a central role in AD pathogenesis and development, it is vital to determine how acupuncture can impact CBF regulation in an AD model. However, the underlying molecular mechanism remains largely elusive. This issue further limits our understanding of MA therapeutic mechanism in AD, and delays the optimization of an MA clinical protocol, since the reduction of CBF, is a sensitive marker to AD early perfusion disorders. Therefore, the moderating effect of MA on the underlying molecular mechanism of CBF regulation requires additional studies.

As an important *in vivo* model in senescence-accelerated proneness, SAMP8 mice present age-related cognitive deterioration (Griñan-Ferré et al., 2016; Akiguchi et al., 2017) and show pathological features similar to the mechanisms responsible for AD pathophysiology, such as oxidative stress, APP overexpression, Aβ deposition and tau phosphorylation (Li et al., 2013; Bayod et al., 2014; Cheng et al., 2014). SAMP8 is a good animal model to investigate the fundamental mechanisms of age-related learning and memory deficits and is widely used for studying early neurodegenerative changes, associated with AD (Butterfield and Poon, 2005; Manich et al., 2011; Griñán-Ferré et al., 2018). This study assessed changes in SAMP8 mice hippocampal neurovascular pathway and compared them to the normal aging process in the control senescence-accelerated resistant 1 (SAMR1) mice (Takeda, 2009). Using the Morris water maze (MWM) test, immunofluorescence (IF) staining, western blot (WB), and liquid chromatography-tandem mass spectrometry (LC-MS/MS), we aimed at elucidating the effect of MA on the astrocytes PLA_2_-AA pathway. To the best of our knowledge, this is the first study, that focused on the underlying mechanism of CBF response after MA treatment in AD. Our data greatly contribute to unraveling the scientific basis of MA in AD treatment and further identify the effectiveness of MA in improving cognitive ability.

## Materials and Methods

### Experimental Animals

Male SAMP8 and male SAMR1 mice were purchased from the Zhi Shan (Beijing) Academy of Medical Science and tested by Chinese Academy of Medical Sciences [Animal Lot: SCXK (Jing) 2014-0011]. Both types of mice weighed 30.0 ± 2.0 g and were 8 months old. The animals were housed in the Experimental Animal Center of Beijing University of Chinese Medicine at a controlled temperature (24 ± 2°C) and under a 12-h dark/light cycle, with sterile drinking water and a standard pellet diet available *ad libitum*. All mice were acclimatized to the environment for 7 days prior to experimentation. Efforts were made to minimize the number of animal uses and the suffering of the experimental animals.

### Animal Grouping and Intervention

**FIGURE 1:**
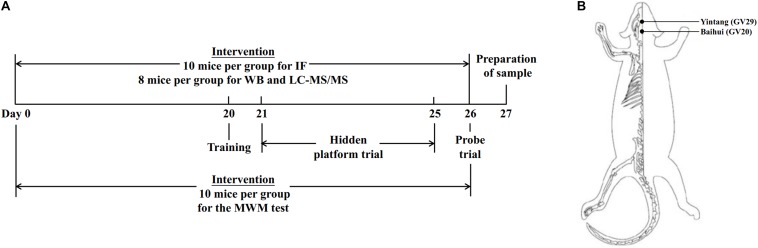
Timeline of experimental design and mouse acupoint locations. **(A)** The timeline of experimental design. **(B)** Illustrated diagram of GV29 and GV20 in the mouse. GV20 is at the midpoint between the auricular apices, and GV29 is located midway between the medial ends of the two eyebrows.

Eighty-four SAMP8 mice were divided into three groups (*n* = 28 per group): the SAMP8 control (Pc) group, the SAMP8 manual acupuncture (Pm) group and the SAMP8 donepezil (Pd) group. Twenty-eight SAMR1 mice were used as the control group (Rc). The timeline of the experimental design and the mouse applied acupoints locations are presented in Figure 1.

In the Pm group, the mice were immobilized in mouse bags. Disposable sterile acupuncture needles (0.25 mm × 13 mm) (Beijing Zhongyan Taihe Medicine Company, Ltd) were used. MA at Baihui (GV20) and Yintang (GV29) was applied for 20 min, with transverse puncturing at a depth of 2–3 mm. During the MA at GV20 and GV29, twirling manipulation was applied every 5 min for 15 s each time. Each needle was rotated bidirectionally within 90° at a speed of 180°/s. For the Pd group, donepezil hydrochloride tablets (Eisai China Inc, H20050978) were crushed and dissolved in distilled water and were delivered to mice by oral gavage at a dose of 0.65 μg/g (Geerts et al., 2005; Ding et al., 2017, 2019). The above treatments were administered once a day for 26 consecutive days, but no treatment was carried out in the Pc or Rc groups. The mice in the Rc, Pc, and Pd groups received the same 20 min restriction as the Pm group. The above intervention lasted throughout the Morris water maze test period.

The selection of the acupoints was based on findings from our previous studies; namely, the therapeutic principle of dredging DU meridian and lighting mind (Jiang et al., 2015, 2016, 2018; Cao et al., 2017; Ding et al., 2019). According to the traditional meridian theory, the DU meridian connects to the brain and is closely associated with all mental activities. Therefore, MA at GV20 and GV29, the important acupoints in the DU meridian, has a benign regulative effect on the DU meridian and is conducive to light mind, which is applicable for AD.

### MWM Test

Ten mice in each group were selected randomly for the MWM test on day 21. To assess the ability of spatial learning and memory, the hidden platform trial and probe trial were conducted in order. The MWM we used in this study has been described previously (Ding et al., 2019).

### Hidden Platform Trial

All mice used for the MWM test were made to carry out swim training for 1 min in the maze on day 20 of the intervention. On day 21, the hidden platform trial was performed. The platform was positioned in the middle of the SW quadrants. Mice were given a series of daily trials using a semirandom set of start locations. The four start locations were used with the restriction that one trial each day was from each of the four positions. Each mouse was released from one of four start locations and had 60 s to search for the hidden platform. At the end of each trial, the mouse was placed on the platform or allowed to stay there for 10 s. Four trials per day were performed for 5 consecutive days, with the visual cues kept constant. The escape latency was collected for subsequent analysis.

### Probe Trial

To assess reference memory, the probe trial was conducted on day 26. The platform was removed, and each mouse was placed in the pool once for 60 s. The starting direction farthest from the platform quadrant was used in the hidden platform trial. The swimming distances in the maze were recorded, and the platform crossover number, swimming speed, and percentage of the time and swimming distance in the platform quadrant were analyzed using EthoVision (3.1.16, Noldus).

### IF Staining

On day 27, 10 mice in each group were anesthetized by an intraperitoneal injection of pentobarbital (80 mg/kg body weight) and perfused with 4% paraformaldehyde. The brains were dissected, fixed in 4% paraformaldehyde for 2.5 h, and dehydrated by 20 and 30% sucrose for 24 h each. Frozen 5-μm sections were sliced by a freezing microtome (CM1900, Leica Corporation, Germany) at −20°C. The primary antibodies included the goat polyclonal GFAP antibody (1:100, Abcam, United States), the rabbit polyclonal PLA_2_ antibody (1:100, Abcam, United States), the rabbit monoclonal COX-1 antibody (1:100, Abcam, United States), and the rabbit polyclonal CYP2C23 antibody (1:100, USBio, United States). Donkey anti-goat IgG Alexa Fluor 488 (1:200, Abcam, United States), and donkey anti-rabbit IgG Alexa Fluor 594 (1:200, Abcam, United States) were used as corresponding secondary antibodies. The 4′,6-Diamidino-2-phenylindole dihydrochloride (DAPI, Solarbio, China) was applied for counterstaining. For double immunohistochemical staining, the sections were washed in 0.1 M PBS (pH = 7.4) and blocked in 0.1 M PBS (pH = 7.4) containing 10% normal donkey serum and 0.5% Triton X-100 for 30 min. Next, the sections were treated with GFAP antibody and PLA_2_ antibody and incubated overnight at 4°C. After PBS washes, the sections were exposed to secondary antibodies for 2 h. Subsequently, the sections were washed with PBS and stained with DAPI for 5 min. After washing with PBS, the sections were observed under a confocal laser scanning microscope (FV1200, Olympus Corporation, Japan). Double immunohistochemical staining using the same staining protocol was undertaken to examine the coexpression of GFAP and COX-1, GFAP and CYP2C23.

Identical exposure times and image settings were used for each experiment. For each specimen, six images of the hippocampus were captured at 100× magnification for quantification. All images were analyzed using ImageJ. The astrocytes in the hippocampus were defined based on GFAP and were outlined “freehand.” The mean optical density of the six images was calculated. The mean optical densities of PLA_2_, COX-1, and CYP2C23 in astrocytes of the hippocampus were compared in each group.

### WB Analysis

On day 27, 8 mice in each group were euthanized by an intraperitoneal injection of pentobarbital sodium (150 mg/kg body weight) to harvest their hippocampi. Right hippocampi were used for WB analysis. After homogenate and protein extraction, SDS-PAGE was performed with a 8% separating gel and a 5% stacking gel and transferred to a 0.45 μm PVDF membrane. Membranes were blocked using 5% non-fat milk in Tris-buffered saline supplemented with 0.1% Tween 20. The primary antibody (PLA_2_, 1:500, Abcam, United States; COX-1, 1:1000, Abcam, United States; CYP2C23, 1:1000, USBio, United States; beta-actin, 1:500, Bioss, United States) was added, followed by incubation overnight at 4°C. The secondary antibody (goat polyclonal rabbit IgG antibody-HRP, 1:3000, Bioss, United States) was added before shaking and incubation at room temperature for 1 h. HRP-ECL luminous liquid was added, and X-ray film exposure was completed in a dark room following developing and fixing. After calibrating the control markers, the scanning and analysis were performed by Quantity One. All western blot bands were normalized with their corresponding β-actin expression (loading control), for the appropriate evaluation of protein expressions. The relative expression of PLA_2_, COX-1, and CYP2C23 was compared in each group.

### LC-MS/MS

On day 27, 8 mice in each group were euthanized by an intraperitoneal injection of pentobarbital sodium (150 mg/kg body weight) to harvest their hippocampus. Left hippocampi were used for LC-MS/MS analysis. After being weighed, the tissues were homogenized with 500 μL of methanol (2% formic acid and 0.01 mol/L BHT) spiked with internal standards from Cayman Chemical Co. (Ann Arbor, MI, United States) (5 ng each of PGE2-d_4_ and 8,9-EET-d_11_). After centrifugation (12,000 *g* for 10 min at 4°C), the supernatant was transferred to a new tube. Water (700 μL) and ethyl acetate (1 mL) were added to the supernatant. The sample was mixed vigorously for 2 min and centrifuged for 10 min at 12,000 *g*. The upper organic phase was transferred to a new tube, and the water phase was extracted again. The organic phase was combined and then evaporated to dryness. The dried residue was dissolved in 100 μL of 30% acetonitrile. An ultraperformance liquid chromatograph (ACQUITY, Waters, United States) and hybrid triple quadrupole linear ion trap mass spectrometer (QTRAP 5500, AB Sciex, Foster City, CA, United States) were used. The quantitative method and conditions for LC-MS/MS were consistent with Zhang et al. (2015). All data were processed using MultiQuant v.2.1 (AB Sciex). The contents of AA, PGE_2_, 5,6-EET, 8,9-EET, 11,12-EET, and 14,15-EET were compared between groups.

### Statistical Analysis

The statistical analysis was performed using the SPSS software, version 17.0 (SPSS, Inc., Chicago, IL, United States), and the data are expressed as the mean ± standard deviation. A two-way ANOVA with repeated measures was used to analyze group differences in the hidden platform trial. A one-way ANOVA followed by the LSD multiple-range test was used to analyze group differences in the probe trial and during PLA_2_-AA pathway activation. For the non-normally distributed data or data with heterogeneous variance, the Kruskal–Wallis test was used. The statistical significance was set to *p* < 0.05 and a high statistical significance was set to *p* < 0.01.

## Results

### Effect of MA on Spatial Learning and Memory

**FIGURE 2:**
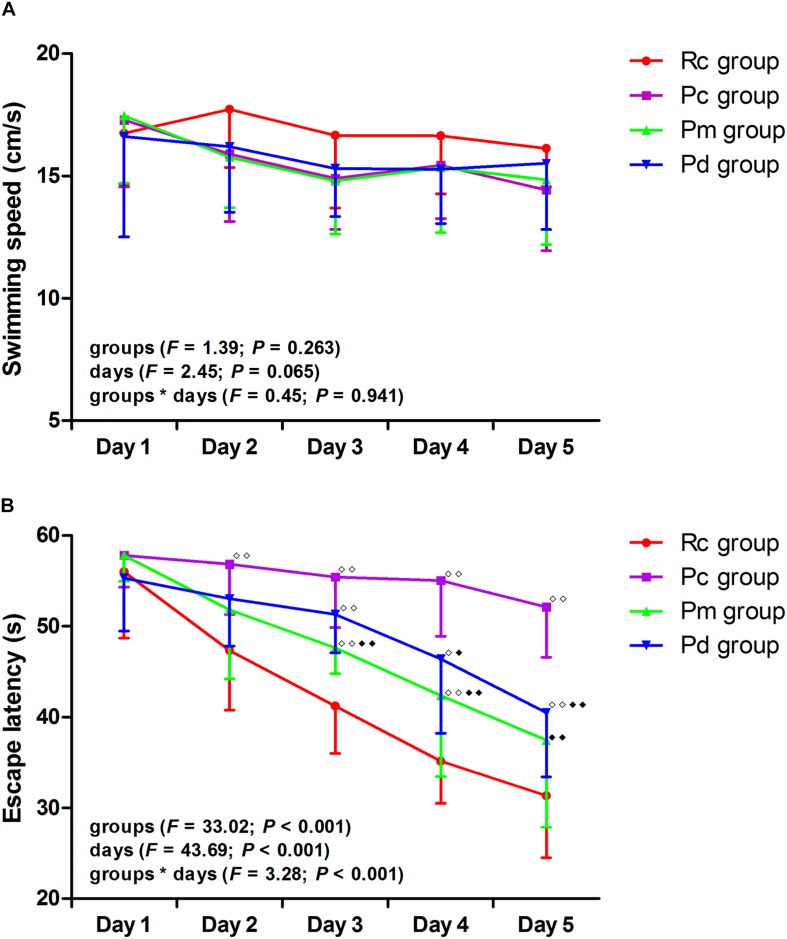
Results of the hidden platform trial in each group (*n* = 10, mean ± SD). **(A,B)** Comparison between the swimming speed and escape latency of all groups. A two-way ANOVA with repeated measures was used. LSD-t was presented in Supplementary Table 1. ^◆◆^*P* < 0.01, ^◆^*P* < 0.05 compared with the Rc group. ^◆◆^*P* < 0.01, ^◆^*P* < 0.05 compared with the Pc group.

**FIGURE 3:**
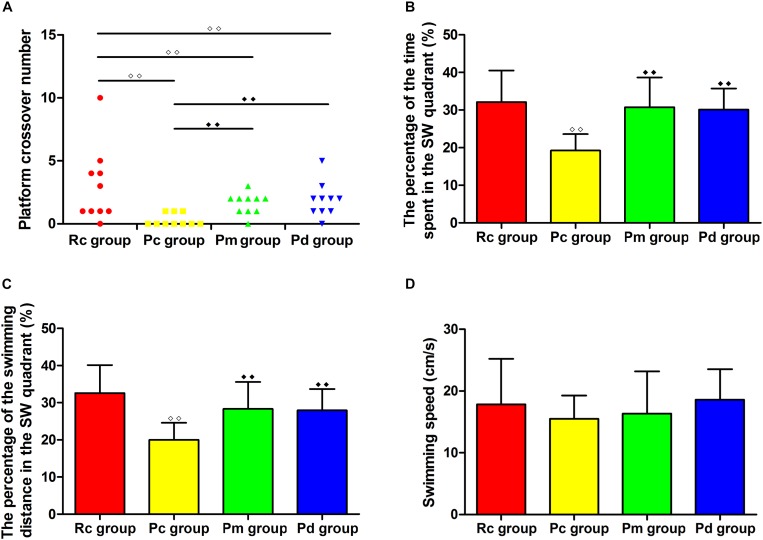
Results of the probe trial in each group (*n* = 10, mean ± SD). **(A–D)** Comparison between the platform crossover numbers, the percentage of time and swimming distances in the SW quadrant, and the swimming speed of all groups. A one-way ANOVA followed by the LSD multiple-range test was used with an exception when comparing the platform crossover numbers, which was analyzed by the Kruskal–Wallis test. LSD-t and Chi-Square are presented in Supplementary Tables 2, 3. ^◆◆^*P* < 0.01, ^◆◆^*P* < 0.01.

The results of the MWM test are presented in Figures 2, 3. In the hidden platform trial, there was no significant difference in the escape latency among groups on day 1. The escape latency of the Rc, Pm, and Pd groups gradually decreased, but the Pc group maintained a high value. From days 2–5, the escape latency was significantly higher in the Pc group than in the Rc group (*p* < 0.01). The escape latency in the Pm and Pd groups was drastically lower than in the Pc group on days 3–5 and days 4–5, respectively (*p* < 0.01 or *p* < 0.05). Compared with that in the Rc group, the escape latency in the Pm and Pd groups was significantly higher on days 3–4 and days 3–5, respectively (*p* < 0.01 or *p* < 0.05). In the probe trial, the platform crossover number and the percentage of time and swimming distance in the platform quadrant in the Pc group, were drastically lower than in the Rc group (*p* < 0.01). In the Pm and Pd groups, the platform crossover number and percentage of time and swimming distance in the platform quadrant, markedly increased compared with those in the Pc group (*p* < 0.01); whereas, the platform crossover number was still lower than those in the Rc group (*p* < 0.01). There were no significant group differences in the swimming speed in the hidden platform or probe trial.

### Effect of MA on the Coexpression of GFAP and PLA_2_, COX-1 and CYP2C23 in the Hippocampus

**FIGURE 4:**
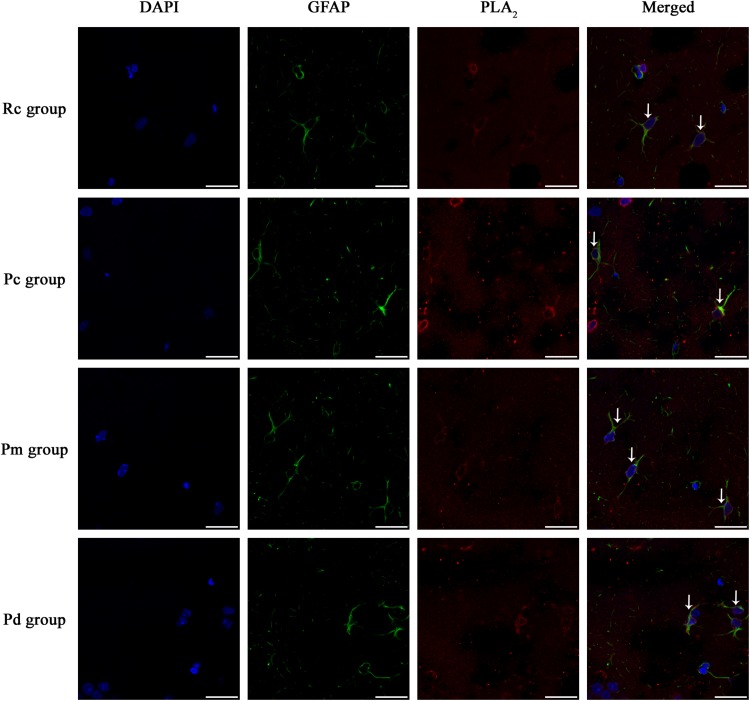
Representative images of IF staining of GFAP (green) and co-expressed PLA_2_ (red) in each group. GFAP is labeled with green fluorescence and the coexpressed PLA_2_ with red fluorescence. The nucleus is labeled with blue fluorescence. The astrocytes showed a polygonal nucleus. In the hippocampus, the positively stained cells are shown with white arrows. Scale bar is 20 μm.

**FIGURE 5:**
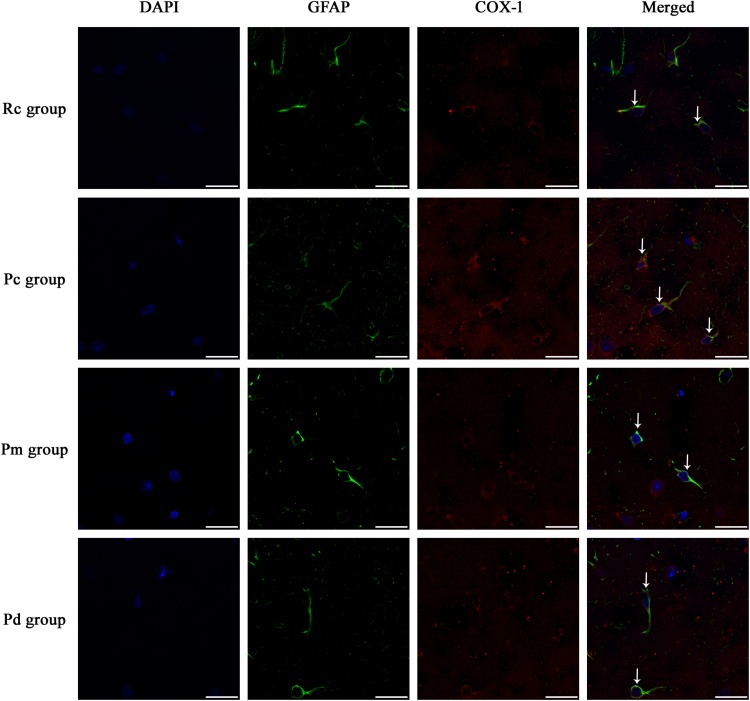
Representative images of IF staining of GFAP (green) and co-expressed COX-1 (red) in each group. GFAP is labeled with green fluorescence and the coexpressed COX-1 with red fluorescence. The nucleus is labeled with blue fluorescence. The astrocytes showed a polygonal nucleus. In the hippocampus, the positively stained cells are shown with white arrows. Scale bar is 20 μm.

**FIGURE 6:**
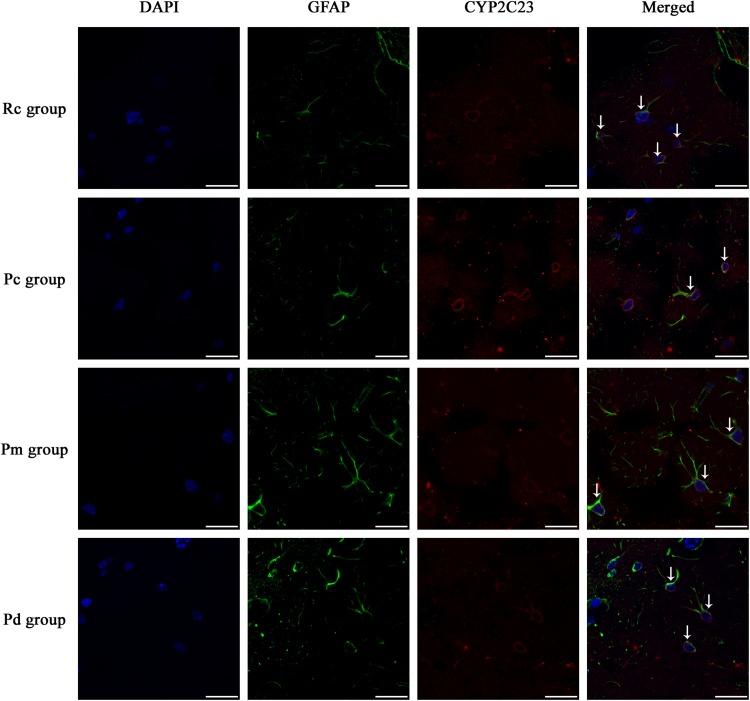
Representative images of IF staining of GFAP (green) and co-expressed CYP2C23 (red) in each group. GFAP is labeled with green fluorescence and the coexpressed CYP2C23 with red fluorescence. The nucleus is labeled with blue fluorescence. The astrocytes showed a polygonal nucleus. In the hippocampus, the positively stained cells are shown with white arrows. Scale bar is 20 μm.

**FIGURE 7:**
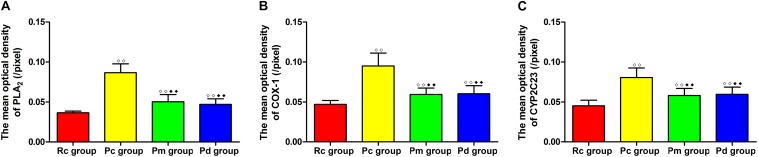
Comparison of the expression of astrocytic PLA_2_, COX-1 and CYP2C23 in the hippocampus between groups (*n* = 10, mean ± SD). **(A–C)** Comparison between the mean optical density of PLA_2_, COX-1 and CYP2C23 of all groups. A one-way ANOVA followed by LSD multiple-range test was used with an exception when comparing COX-1, which was analyzed by the Kruskal–Wallis test. LSD-t and Chi-Square are presented in Supplementary Tables 2, 4. ^◆◆^*P* < 0.01, ^◆◆^*P* < 0.01.

IF staining and quantitative analysis of PLA_2_, COX-1 and CYP2C23 in the hippocampus are presented in Figures 4–7. The astrocytes were scattered in the hippocampus. GFAP was coexpressed with PLA_2_, COX-1 and CYP2C23 in the cytoplasm and processes. The fluorescence intensity of PLA_2_, COX-1 and CYP2C23, which were coexpressed with GFAP in the Pc group, was higher than that in the Rc group. Compared with that in the Pc group, there was a decrease in the fluorescence intensity of PLA_2_, COX-1 and CYP2C23 in the Pm and Pd groups. The quantitative analysis showed, that the mean optical density of PLA_2_, COX-1 and CYP2C23 in the Pc, Pm, and Pd groups, was significantly higher than that in the Rc group (*p* < 0.01). The mean optical density of PLA_2_, COX-1 and CYP2C23 in the Pm and Pd groups was markedly lower than that in the Pc group (*p* < 0.01).

### Effect of MA on the Relative Expression of PLA_2_, COX-1 and CYP2C23 in the Hippocampus

**FIGURE 8:**
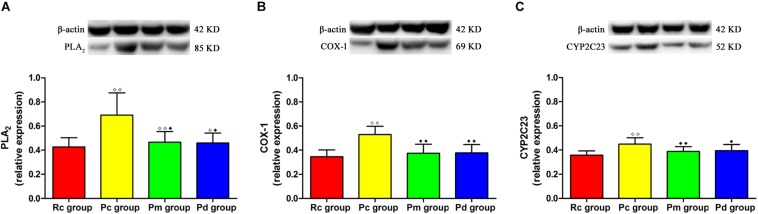
WB results in each group. **(A–C)** Comparison between the relative expression of PLA_2_, COX-1 and CYP2C23 in the hippocampus (*n* = 8, mean ± SD). A one-way ANOVA followed by LSD multiple-range test was used with an exception when comparing PLA_2_, which was analyzed by Kruskal–Wallis test. LSD-t and Chi-Square are presented in Supplementary Tables 2, 5. ^◆◆^*P* < 0.01, ^◆^*P* < 0.05 compared with the Rc group. ^◆◆^*P* < 0.01, ^◆^*P* < 0.05 compared with the Pc group.

The western blotting results of PLA_2_, COX-1 and CYP2C23 protein expressions, in the hippocampus, are presented in Figure 8. The relative expression of PLA_2_, COX-1, and CYP2C23 in the Pc group was significantly higher than that in the Rc group (*p* < 0.01). Compared with that in the Pc group, the relative expression of PLA_2_, COX-1 and CYP2C23 in the Pm and Pd groups decreased drastically (*p* < 0.01 or *p* < 0.05). The relative expression of PLA_2_ in the Pm and Pd groups was higher than that in the Rc group (*p* < 0.01 or *p* < 0.05).

### Effects of MA on the Contents of AA, PGE_2_ and EETs in the Hippocampus

**FIGURE 9:**
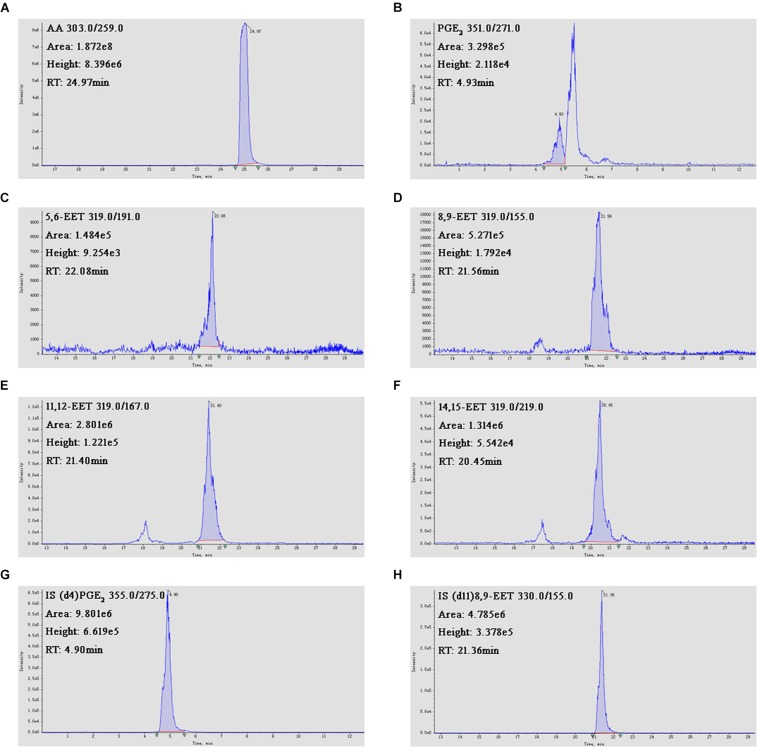
The LC-MS/MS results. **(A–H)** Chromatographs of AA, PGE_2_, 5,6-EET, 8,9-EET, 11,12-EET, 14,15-EET, 8,9-EET-d_11_ and PGE2-d_4_.

**FIGURE 10:**
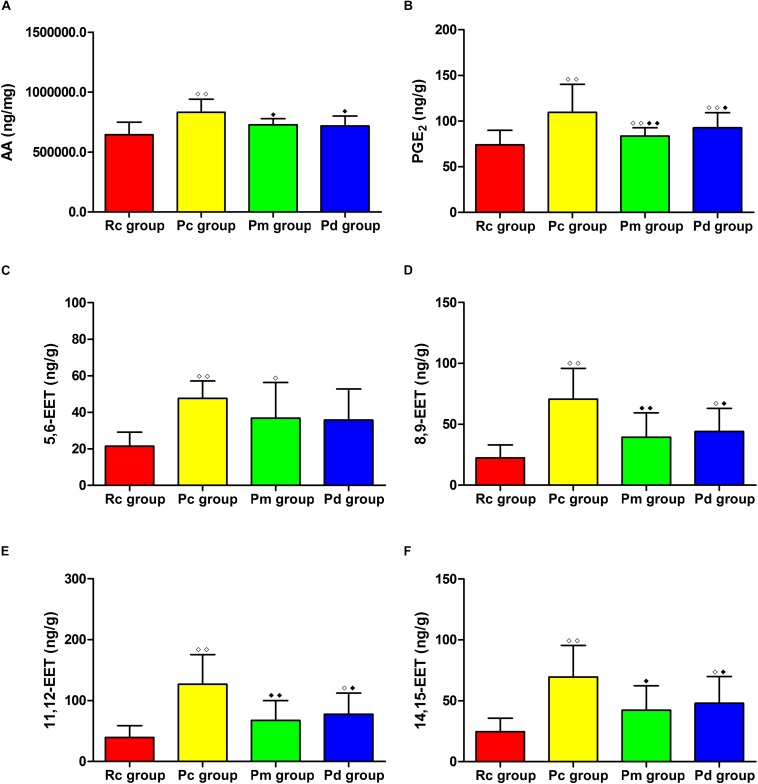
Comparison between the contents of AA, PGE_2_ and EETs between groups (*n* = 8, mean ± SD). **(A–H)** Comparison between the contents of AA, PLA_2_, 5,6-EET, 8,9-EET, 11,12-EET and 14,15-EET in the hippocampus. A one-way ANOVA followed by LSD multiple-range test was used with an exception when comparing PGE_2_, which was analyzed by Kruskal–Wallis test. LSD-t and Chi-Square are presented in Supplementary Tables 2, 6. ^◆◆^*P* < 0.01, ^◆^*P* < 0.05 compared with the Rc group. ^◆◆^*P* < 0.01, ^◆^*P* < 0.05 compared with the Pc group.

The LC-MS/MS results of AA, PGE_2_ and EETs are presented in Figures 9, 10. Compared with those in the Rc group, the contents of AA, PGE_2_, 5,6-EET, 8,9-EET, 11,12-EET and 14,15-EET in the Pc group significantly increased (*p* < 0.01). The contents of AA, PGE_2_, 8,9-EET, 11,12-EET and 14,15-EET in the Pm and Pd group were markedly lower than those in the Pc group (*p* < 0.01 or *p* < 0.05). The contents of PGE_2_ and 5,6-EET in the Pm group and PGE_2_, 8,9-EET, 11,12-EET and 14,15-EET in the Pd group, were drastically higher than those in the Rc group (*p* < 0.01 or *p* < 0.05).

## Discussion

The abnormality of the cerebrovascular function is one of the basic pathological changes and a key point in AD pathogenesis. Acupuncture plays an active role in the clinical efforts against AD and our previous study showed that MA can effectively enhance CBF in the prefrontal lobes and hippocampi of SAMP8 mice (Ding et al., 2019). However, the underlying molecular mechanism remains largely elusive. In this study, we aimed to elucidate the effect of MA on the astrocytes PLA_2_-AA pathway and on the cognitive ability of SAMP8 mice.

As a classic behavioral experiment, the MWM test has been widely used to evaluate spatial learning and memory. Our results showed that there were no significant differences in swimming speed among the groups in the hidden platform or probe trials, suggesting identical swimming ability. Swimming speed deviations from the norm on the behavioral test were excluded. In the Pc group, the behavioral results showed that the escape latency on days 2–5 of the hidden platform trial was significantly higher than in the Rc group (*p* < 0.01). The platform crossover number and the percentage of time and swimming distance in the platform quadrant were drastically decreased (*p* < 0.01). These results demonstrate that the mice in the Pc group suffered a marked decline in memory and learning abilities, which is in accordance with what is observed with AD pathological changes. Both MA and donepezil significantly increased the platform crossover number and percentage of time and swimming distance in the platform quadrant; while, decreasing the escape latency (*p* < 0.01). These observations indicate, that the equal effects of MA and donepezil, can effectively improve the learning and memory abilities of SAMP8 mice. It is worth noting that, in the hidden platform trial; while, the most notable difference in the escape latency between the Pc and Pm group occurred on day 3 (*p* < 0.01), similar changes appeared on day 4 for the Pd group (p < 0.05). Besides, the escape latency in the Pd group was still drastically higher than that of the Rc group on days 3–5 (*p* < 0.01 or *p* < 0.05). These results demonstrate, that MA tends to have a greater effect in improving SMAP8 mice spatial learning ability, in accordance with our previous study (Ding et al., 2019). In this study, the behavioral experiments using the MWM test further confirmed the effectiveness of MA in improving AD cognitive ability. Additionally, the results suggest, that the mechanism by which MA counteracts AD, is different from that of donepezil. We speculate, that this difference, is related to the multitarget nature of acupuncture (Cao et al., 2016), which manifests anti-inflammatory (Ding et al., 2017), anti-oxidative (Liu et al., 2013; Sutalangka et al., 2013), anti-apoptotic (Guo et al., 2015) and anti-Aβ effects (Wang et al., 2016; Liu et al., 2017; Zhang M. et al., 2017) in AD. These effects meet the need for a multimodal AD therapy (Ubhi and Masliah, 2013; Ibrahim and Gabr, 2019). Considering of the advantages of MA, such as safety and convenience, additional studies of MA effects on AD should be performed. In this study, the technical details, such as acupoints selection, manipulation and treatment arrangement, provide a valid reference for clinical researchers.

This study also shows the limitation of MA, as manifested in the significant differences in cognitive ability between Pm and Rc groups. SAMP8 mice present age-related deteriorations in behavior, physiology, neuropathology, and neurochemistry (Takeda, 2009). These mice exhibit Aβ deposition in the hippocampus, as early as 6 months, and that progresses with age. In contrast, this process does not start in SAMR1 mice until 15 months (Del Valle et al., 2010). Thus, we speculate that the limited beneficial effects of MA on cognitive ability were due to SAMP8 mice advanced age. Furthermore, our data suggest, that MA interventions, should be initiated at an early stage to better elucidate the characteristics of MA anti-AD effect. On the other hand, our results reconfirm, that for a chronic neurodegenerative disease with complex pathogenesis, such as AD, early intervention is of great significance for effective disease control and good prognosis, and which aligns with the common view on AD treatment (Khan, 2018).

In this study, COX-1, an astrocyte constitutive enzyme, that is closely associated with resting blood flow (Takano et al., 2006; Rosenegger et al., 2015) and CYP2C23, a key enzyme in the biosynthesis of EETs (Capdevila et al., 2015), had significantly higher mean optical densities in the Pc group when compared to the Rc group (*p* < 0.01). These results were similar for the astrocytic PLA_2_ and the contents of AA, PGE_2_ and EETs, which were also significantly higher in the Pc group compared to those in the Rc group (*p* < 0.01). These findings were consistent with previous studies (Esposito et al., 2008; Sanchez-Mejia et al., 2008; Sanchez-Mejia and Mucke, 2010), that demonstrated an upregulation of the astrocytic PLA_2_-AA pathway in the hippocampus of the Pc group, which involved an increase in the expression of COX-1, CYP2C23 and the contents of PGE_2_ and EETs. The changes corresponded to a the neurovascular response to a lower CBF, that was caused by the deposition of Aβ in AD (Hamel, 2015; Kimbrough et al., 2015), and which also suggested an over activation of the PLA_2_-AA pathway in the Pc group. When combining these results with the previously reported lower CBF in SAMP8 mice compared to SAMR1 mice (Ding et al., 2019), the results show that the compensatory neurovascular response fails to enhance CBF, demonstrating a cerebral neurovascular dysfunction in SAMP8 mice. One possible reason for this failure is the structural and functional impairments of the cerebral microvasculature, that are induced by Aβ deposition, and which are important mechanisms for AD cerebrovascular damage (Dorr et al., 2012; Kimbrough et al., 2015; Sisante et al., 2019). The cerebral amyloid angiopathy, caused by the deposition of Aβ in the wall of cerebral blood vessels, not only destroys the cerebrovascular structure, but also impairs the neurovascular coupling (Lai et al., 2015; Giannoni et al., 2016; Yan et al., 2017; Hecht et al., 2018). Generally, the results suggest that under such circumstances, therapeutic strategies that attempt to enhance CBF through vasodilation, are less feasible. Therefore, early intervention and protection of the cerebral microvascular structure and function, as far as possible, are very important for delaying the pathological process and thus, support AD clinical treatment.

Both MA and donepezil can reverse such changes and drastically downregulate PLA_2_, COX-1, CYP2C23 and the contents of AA, PGE_2_ and EETs in the hippocampus (*p* < 0.01 or *p* < 0.05). The PLA_2_-AA pathway and AA metabolism were at normal levels in the Pm and Pd groups. Aside from their different effects on EETs, there were no notable differences between the moderating effects of MA and donepezil on PLA_2_, AA downstream metabolic pathway and related proteins or metabolites between the Pm and Pd groups. Our results showed that the contents of 8,9-EET, 11,12-EET, and 14,15-EET in the Pd group significantly increased compared with those in the Rc group (*p* < 0.05). This change did not occur in the Pm group, indicating that the effect of MA on EETs has a better effectiveness. Compared with the Pc group, the Pm and Pd groups exhibited an inhibition of the vasodilatory pathways (*p* < 0.01 or *p* < 0.05). Based on our previous report on the beneficial effects of MA and donepezil on CBF, it is clear that MA enhances CBF through ‘abnormal’ vasoconstriction rather than typical vasodilatation, which is the regular mean for a normal physiological state. This result did not conform with previous studies that demonstrated an increased blood flow that was associated with blood flow velocity and vasodilatation (Stoner et al., 2004; Domoki et al., 2012). Since CBF is defined as the CBF velocity × cross-sectional area, the paradoxical relationship between the ‘abnormal’ vasoconstriction found in this study and the increase in CBF suggests that the regulation of the CBF velocity may be a reason for the beneficial effect of MA on CBF. In particular, the inhibition of the vasodilatory pathway may result in increasing the arterial tone, which eventually promotes the blood flow velocities. Our results also suggest that conduit arteries, which can dilate or constrict depending of the arterial tone, may be potential targets in the CBF regulation by MA, as arterioles tend to be responsible for the hemodynamic resistance. In summary, we speculate that changes of the arterial tone in conduit arteries, induced by the inhibition of the vasodilatory pathway, may be a reason for the beneficial effect of MA on CBF. It is noteworthy that this kind of vasoconstriction is an ‘abnormal’ intervention mode under the pathological status of AD.

There are some limitations to this study. First, the sample size in the behavioral test was small. Since a difference between MA and donepezil in improving cognitive ability was observed, further studies should clarify the characteristics of MA. Additionally, a behavioral test should be performed before the intervention to better explain the effect of MA. This study suggests that the benign effect of MA on CBF may be based on the increase in CBF velocity, induced by the changes of arterial tone. Second, the PLA_2_ activity and the access to its phospholipid substrate is increased by Ca^2+^ binding to its C2 domain and phosphorylation by the ERK/MAPK kinases (Sanchez-Mejia and Mucke, 2010). Therefore, the effect of MA on Ca^2+^, the phosphorylation of PLA_2_ and related kinases warrant further investigations. Besides, this study revealed that MA has a negative regulatory effect on the PLA_2_-AA pathway, and we speculate that the regulation of the CBF velocity may be a reason for the beneficial effect of MA on CBF. Combined with our previous study (Ding et al., 2019), the results suggest a benign regulatory effect of MA on the structure and function of cerebral vessels. Future studies should observe and further confirm the modulatory effect of MA on the morphology of cerebral vessels and hemodynamics through histological, biochemical and *in vivo* imaging techniques, such as two-photon microscopy (Song et al., 2017), transcranial Doppler ultrasound (Liu et al., 2013) and photoacoustic microscope (Ning et al., 2015). Finally, the limited effects of MA and donepezil on cognitive ability demonstrated that there is still potential for optimization. Earlier MA intervention, longer duration of MA and stronger doses of donepezil should be encouraged.

In summary, this study validated the authenticity and reliability of using MA to improve cognitive ability, with MA tending to have a greater effect in improving spatial learning ability in SMAP8 mice. Additionally, this study revealed the underlying mechanism of the beneficial effects of MA on CBF and found that MA has a negative regulatory effect on the PLA_2_-AA pathway, as reflected in its downregulation of PLA_2_, COX-1, CYP2C23, and the contents of AA, PGE_2_, and EETs in SAMP8 mice hippocampi. We propose that the changes of arterial tone, induced by the inhibition of vasodilatory pathway, might be a reason for the beneficial effect of MA on CBF. Further *in vivo* studies should be encouraged to observe and confirm the modulatory effect of MA on cerebral vessels and hemodynamics.

## Data Availability Statement

All datasets generated for this study are included in the article/Supplementary Material.

## Ethics Statement

All experimental procedures complied with the guidelines of the Principles of Laboratory Animal Care formulated by the National Institute of Health and the legislation of the People’s Republic of China for the use and care of laboratory animals. The experimental protocols were approved by the Medicine and Animal Ethics Committee of the Beijing University of Chinese Medicine.

## Author Contributions

ND: experimental design, data analysis, and manuscript preparation. HT and SW: data collection. JJ and ZL: experimental design. All authors contributed to drafting the manuscript and have read and approved the final manuscript.

## Conflict of Interest

The authors declare that the research was conducted in the absence of any commercial or financial relationships that could be construed as a potential conflict of interest.
