# Supplementary material for: Benign Regulation of the Astrocytic Phospholipase A2-Arachidonic Acid Pathway: The Underlying Mechanism of the Beneficial Effects of Manual Acupuncture on CBF
Source: Front Neurosci. 2020 Feb 4;13:1354. doi: 10.3389/fnins.2019.01354 (PMC7054756; doi:10.3389/fnins.2019.01354)
Supplement: Supplementary file 1 [file Table_1.DOC]

**SUPPLEMENTARY TABLE 1 | The LSD-t of escape latency in hidden platform trial (t, P).**

| **Groups** | **Day 2** | **Day 3** | **Day 4** | **Day 5** |
| --- | --- | --- | --- | --- |
| Rc | - | - | - | - |
| Pc | (-3.38; 0.002) | (-6.90; < 0.001) | (-6.20; < 0.001) | (-6.29; < 0.001) |
| Pm | - | Pm-Rc (-3.10; 0.004)  Pm-Pc (3.80; 0.001) | Pm-Rc (-2.25; 0.031)  Pm-Pc (3.96; < 0.001) | (4.44; < 0.001) |
| Pd | - | (-4.90; < 0.001) | Pd-Rc (-3.50; 0.001)  Pd-Pc (2.70; 0.011) | Pd-Rc (-2.77, 0.009)  Pd-Pc (3.52; 0.001) |

**SUPPLEMENTARY TABLE 2 | The Chi-Square (Chi-Square, P).**

| **Groups** | **Platform crossover numbers** | **COX-1 in IF** | **PLA_2_ in WB** | **PGE_2_ in LC-MS/MS** |
| --- | --- | --- | --- | --- |
| Rc | - | - | - | - |
| Pc | (8.99; 0.003) | (14.30; < 0.001) | (7.47; 0.006) | (10.60; 0.001) |
| Pm | Pm-Rc (12.39; 0.002)  Pm-Pc (9.71; 0.002) | Pm-Rc (23.60; < 0.001)  Pm-Pc (14.29; < 0.001) | Pm-Rc (9.56; 0.008)  Pm-Pc (5.34; 0.021) | Pm-Rc (13.72; 0.001)  Pm-Pc (8.65; 0.003) |
| Pd | Pd-Rc (14.19; 0.003)  Pd-Pc (12.84; 0.002) | Pd-Rc (28.84; < 0.001)  Pd-Pc (18.98; < 0.001) | Pd-Rc (10.80; 0.013)  Pd-Pc (7.63; 0.022) | Pd-Rc (15.35; 0.002)  Pd-Pc (8.82; 0.012) |

**SUPPLEMENTARY TABLE 3 | The LSD-t of the percentage of time and swimming distances in the SW quadrant in probe trial (t, P).**

| **Groups** | **The percentage of time spent in the SW quadrant** | **The percentage of swimming distances in the SW quadrant** |
| --- | --- | --- |
| Rc | - | - |
| Pc | (4.25; < 0.001) | (4.43; < 0.001) |
| Pm | (-2.79; 0.001) | (-2.93; 0.006) |
| Pd | (-3.60; 0.001) | (-2.80; 0.008) |

**SUPPLEMENTARY TABLE 4 | The LSD-t of the expression of PLA2 and CYP2C23 in IF (t, P).**

| **Groups** | **PLA_2_** | **CYP2C23** |
| --- | --- | --- |
| Rc | - | - |
| Pc | (-14.38; < 0.001) | (-8.34; < 0.001) |
| Pm | Pm-Rc (-3.95; < 0.001)  Pm-Pc (10.44; < 0.001) | Pm-Rc (-3.04; 0.004)  Pm-Pc (5.30; < 0.001) |
| Pd | Pd-Rc (-3.02; 0.005)  Pd-Pc (11.36; < 0.001) | Pd-Rc (-3.34; 0.002)  Pd-Pc (5.00; < 0.001) |

**SUPPLEMENTARY TABLE 5 | The LSD-t of the expression of COX-1 and CYP2C23 in WB (t, P).**

| **Groups** | **COX-1** | **CYP2C23** |
| --- | --- | --- |
| Rc | - | - |
| Pc | (-4.13; < 0.001) | (-5.44; < 0.001) |
| Pm | (2.79; < 0.001) | (4.59; 0.009) |
| Pd | (2.46; < 0.001) | (4.52; 0.020) |

**SUPPLEMENTARY TABLE 6 | The LSD-t of the contents of AA and EETs (t, P).**

| **Groups** | **AA** | **5,6-EET** | **8,9-EET** |  | **11,12-EET** | **14,15-EET** |
| --- | --- | --- | --- | --- | --- | --- |
| Rc | - | - | - | | - | - |
| Pc | (-4.16; < 0.001) | (-3.66; 0.001) | (-4.96; < 0.001) | | (-4.93; < 0.001) | (-4.38; < 0.001) |
| Pm | (2.29; < 0.029) | (-2.14; 0.041) | (3.22; < 0.003) | | (3.36; 0.002) | (-2.67; 0.013) |
| Pd | (2.50; < 0.018) |  | Pd-Rc (-2.23; 0.034)  Pd-Pc (2.73; 0.011) | | Pd-Rc (-2.16; 0.039)  Pd-Pc (2.77; 0.010) | Pd-Rc (-2.27; 0.031)  Pd-Pc (2.11; 0.044) |
